# Maternal Mercury Exposure, Season of Conception and Adverse Birth Outcomes in an Urban Immigrant Community in Brooklyn, New York, U.S.A.

**DOI:** 10.3390/ijerph110808414

**Published:** 2014-08-18

**Authors:** Cynthia J. Bashore, Laura A. Geer, Xin He, Robin Puett, Patrick J. Parsons, Christopher D. Palmer, Amy J. Steuerwald, Ovadia Abulafia, Mudar Dalloul, Amir Sapkota

**Affiliations:** 1 Maryland Institute for Applied Environmental Health, University of Maryland School of Public Health, College Park, Room 2234F, College Park, MD 20742–2611, USA; E-Mails: cbashore@umd.edu (C.J.B.); rpuett@umd.edu (R.P.); amirsap@umd.edu (A.S.); 2 Department of Environmental and Occupational Health Sciences, Downstate School of Public Health, State University of New York, Box 43,450 Clarkson Ave., Brooklyn, NY 11203–2533, USA; 3 Department of Epidemiology and Biostatistics, University of Maryland College Park School of Public Health, 2234H SPH Building, College Park, MD 20742–2611, USA; E-Mail: xinhe@umd.edu; 4 Laboratory of Inorganic and Nuclear Chemistry, Wadsworth Center, Department of Health, New York State University, Albany, NY 12201–0509, USA; E-Mails: pparsons@wadsworth.org (P.J.P.); palmer@wadsworth.org (C.D.P.); asteuerw@wadsworth.org (A.J.S.); 5 Department of Environmental Health Sciences, University at Albany School of Public Health, Albany, NY 12201, USA; 6 Department of Obstetrics and Gynecology, State University of New York Downstate Medical Center, 445 Lenox Road, Brooklyn, NY 11203, USA; E-Mails: ovadia.abulafia@downstate.edu (O.A.); mudar.dalloul@downstate.edu (M.D.)

**Keywords:** preterm birth, low birth weight, mercury, season of conception, urban immigrant

## Abstract

Adverse birth outcomes including preterm birth (PTB: <37 weeks gestation) and low birth weight (LBW: <2500 g) can result in severe infant morbidity and mortality. In the United States, there are racial and ethnic differences in the prevalence of PTB and LBW. We investigated the association between PTB and LBW with prenatal mercury (Hg) exposure and season of conception in an urban immigrant community in Brooklyn, New York. We recruited 191 pregnant women aged 18–45 in a Brooklyn Prenatal Clinic and followed them until delivery. Urine specimens were collected from the participants during the 6th to 9th month of pregnancy. Cord blood specimens and neonate anthropometric data were collected at birth. We used multivariate logistic regression models to investigate the odds of LBW or PTB with either maternal urinary mercury or neonate cord blood mercury. We used linear regression models to investigate the association between continuous anthropometric outcomes and maternal urinary mercury or neonate cord blood mercury. We also examined the association between LBW and PTB and the season that pregnancy began. Results showed higher rates of PTB and LBW in this cohort of women compared to other studies. Pregnancies beginning in winter (December, January, February) were at increased odds of LBW births compared with births from pregnancies that began in all other months (OR7.52 [95% CI 1.65, 34.29]). We observed no association between maternal exposure to Hg, and either LBW or PTB. The apparent lack of association is consistent with other studies. Further examination of seasonal association with LBW is warranted.

## 1. Introduction

Adverse birth outcomes including preterm birth (PTB: <37 weeks gestation) and low birth weight (LBW: <2500 g) result in severe infant morbidity and mortality [1,2]. Risk factors for PTB include increased maternal age, black race, infections, toxicant exposure (e.g., cigarette smoke and illicit drug use), stress, over/underweight, underlying maternal health conditions (hypertension, obesity, and diabetes), clinical depression, and multiple gestations and prior PTB [3]. Genetic, demographic, and socioeconomic factors, pre-existing medical conditions, complications during pregnancy, inadequacies in prenatal care, as well as consumption of tobacco, caffeine, illicit drugs and alcohol are associated with the risk of LBW [4,5,6,7,8]. Prenatal exposures to pollutants such as organochlorines, formaldehyde, nitrogen dioxide (NO_2_), and particulate matter (PM_2.5_, PM_10_) have been shown to be associated with altered fetal growth or PTB [8,9,10,11,12,13,14].

In the United States, there are racial and ethnic differences in the prevalence of LBW and infant mortality [2,15,16,17,18,19]. For example, non-Hispanic blacks have the highest PTB rates (15%–18%) compared with other racial ethnic groups [3]. Similarly, for immigrant women, maternal country of birth can predict adverse birth outcomes [20]. In some cases, recent immigrants had lower rates of adverse birth outcomes [15,21,22], but this advantage decreased with increasing years of residence and acculturation [23]. A plausible explanation for this observation may be changes in lifestyle, including dietary habits. For example, in terms of fish consumption, various recent immigrant groups such as Chinese and Caribbean’s have been shown to consume fish more frequently. Larger meal size could also contribute to higher mercury exposures [24].

Fish consumption is associated with an increased exposure to mercury (Hg), and specifically methyl mercury (MeHg) which has the potential to cross the placenta and exert its toxic effects on the developing fetus. *In utero* Hg exposure has been linked to fetal malformations and decreased fetal survival in high-dose animal toxicology studies. One proposed mechanism is oxidative stress on the fetus [25]. Neuro-developmental disorders resulting from prenatal exposure to Hg have been documented previously [26,27,28,29,30], however the impact of Hg and frequency of fish consumption on adverse birth outcomes is not clearly defined or understood [31,32]. There is limited evidence of Hg effects on fetal growth and birth outcomes, and specifically birthweight [33,34] and some studies demonstrate no effects [35,36,37]. Others have reported an inverse association between Hg exposure and neonates’ attained weight during the first 24 months of life, suggesting that effects may extend beyond parturition [32]. Fish consumption could serve as a proxy for exposure to other bioaccumulative contaminants (such as PCBs) that could have adverse impacts on birthweight [38]. Alternately, the positive benefits of omega-3 fatty acids can be a proxy for healthy behavior/nutritional status in general that could impart a positive impact on birthweight [38].

Studies have shown that, in addition to environmental and behavioral risk factors, season of conception and birth have been associated with adverse birth outcomes [39,40]. Temperature, air pollution, and increased industrial activity, as well as nutritional habits and food intake surrounding harvest periods or times of low food availability are examples of exposures that vary seasonally and may influence birth outcomes [39,41,42,43]. Thus season of conception or birth can be a proxy for exposures that vary temporally throughout the year. Seasonal association with PTB and LBW varies according to geographic latitude, national economic development status, predominant infectious diseases [39], and Vitamin D exposure [44]. Studies of racial ethnic groups in New York City have reported increased odds of LBW and PTB in some racial ethnic groups including immigrant communities such as Puerto Ricans and other Latino groups as well as in infants of mothers from the Sub-Saharan African region [19]. In this study, we examined the association of prenatal Hg exposure and season of conception with PTB and LBW in a high-risk population of African-American, Caribbean and West Indian women in an urban immigrant community in Brooklyn, New York. We further examined the association of prenatal Hg exposure with neonate anthropometric data.

## 2. Materials and Methods

### 2.1. Study Population and Questionnaire Assessment

A prospective study of pregnant women was conducted at the University Hospital of Brooklyn’s Prenatal Clinic to investigate the association between maternal exposure to several pollutants and risk of adverse birth outcomes. The full study details are described elsewhere [45]. Briefly, a convenience sample of 191 pregnant women between the ages of 18 and 45 were recruited during the 6–9th month of pregnancy from October 2007 to December 2009. Data were collected with a pretested, culturally appropriate questionnaire designed in cooperation with local community groups, including Caribbean physicians. The questionnaire assessed demographic and lifestyle factors that may contribute to Hg exposure such as dietary factors, use of Hg-containing products in the home, use of skin-lightening creams, occupational exposures, number of dental amalgams, and use of Hg in folk medicine practices. Results of the assessment of environmental risk factors for Hg exposure are described in the parent study [45]. Fish and shellfish consumption was estimated by showing participants a pictorial chart of various fish and shellfish species and asking the women about frequency of consumption and type of fish consumed during the current pregnancy. All women were provided with educational materials that described environmental sources of Hg and methods for avoiding Hg exposure.

### 2.2. Collection and Measurement of Maternal Urinary and Neonate Cord Blood Hg

During the 6th to 9th month of pregnancy participants provided a “spot” urine specimen for Hg and creatinine measurement. At delivery a physician or a midwife collected a neonatal cord blood specimen for total Hg determination. Chart review at birth provided demographic data including mother’s age, country of birth and date of immigration, race and ethnic origin, marital status and education level. The initial study protocol was approved by the SUNY Downstate Institutional Review Board (IRB) and by the New York State Department of Health’s IRB. An informed consent was received and signed by participants prior to participation.

Urine specimens were collected and analyzed for creatinine at SUNY Downstate and for total Hg by the Trace Elements Section of the Laboratory of Inorganic and Nuclear Chemistry, Wadsworth Center, NYS Department of Health (DOH) using methods described previously [45]. Urine collected at SUNY was separated onsite into 2 mL and 10 mL aliquots. To adjust for diurnal variations in urine dilution, the 2 mL aliquot of urine was measured at SUNY for creatinine using the Alkaline Picrate Method and a Beckman Olympus Analyzer, Model AU-2700 (Beckman Coulter, Inc., Brea, CA, USA). The 10 mL aliquot was transferred into a trace element collection tube containing Triton X-100 and sulfamic acid preservative to prevent losses of inorganic Hg. At the NYS DOH, total urinary Hg was determined using a Perkin Elmer Model DRC II (Perkin Elmer Life Sciences, Shelton CT, USA) inductively coupled plasma–mass spectrometer (ICP-MS) as previously described [45]. Cord blood specimens were analyzed for total Hg by ICP-MS, as described previously [46]. During analysis, it was noted that some of the cord blood specimens developed fibrin clots, which is quite common for cord blood. In such instances, the blood specimens were sonicated for one hour in an ultrasonic-bath, which was found to be sufficient to dissipate the micro-clots, and permit the analysis to proceed. The method limits of detection (LOD) were 0.24 and 0.09 µg/L cord blood Hg and urinary Hg, respectively [45]. All specimens that were found to be below the detection limit were assigned ½ LOD values.

### 2.3. Statistical Analysis

The study database included 191 mother-neonate pairs. For the purpose of this study, data analysis was restricted to singleton births (*n =* 187). Observations that included only gender (*n =* 2), contained no infant data (*n =* 20), or did not include data for the number of weeks gestation (*n =* 6), neonate birth weight (*n =* 1), and either cord blood Hg or urine Hg and urine creatinine (*n =* 1) were excluded, resulting in a final database of 159 singleton births.

Creatinine-corrected values for urine Hg expressed in units of µg Hg per gram creatinine (µg/g) were used in all regression analyses. In linear regression models, appropriate transformations were applied to meet the normality assumption. For instance, cord blood and creatinine-corrected urine Hg were natural log transformed, neonate head circumference was raised to the third power, and neonate length was squared. Three outliers, one extremely preterm and small neonate (27 weeks gestation, 33 cm length and 1105 g) and two other neonates (36 weeks, 54 cm length and 4355 g, and 39 weeks, 54 cm and 4570 g were removed from the birth weight and head circumference linear regressions, as such values were deemed beyond the range of possible values.

The Kruskal-Wallis test was used to determine if the distributions of cord blood and/or maternal urinary Hg levels differed by LBW, PTB and/or maternal race/ethnicity. We used univariate linear regression to test the associations between maternal characteristics and birth weight, head circumference, and infant length, and multivariate linear regression to investigate the association of neonate cord blood or maternal urinary Hg level with birth weight, head circumference, and body length. Models were adjusted for previously identified risk factors impacting birthweight including maternal age, educational attainment, race/ethnicity, living with partner/spouse [3], and in the case of birthweight models, term of birth. Individual cell size was limited and thus we were unable to analyze dietary intake of specific predatory fish species. The outcome measures included in the logistic regression (LBW, PTB) were dichotomous, while those used in the linear regression (birthweight, head circumference) were continuous. In addition, age, education, and race were coded as categorical variables, while both cord blood and urinary Hg (including corrected for creatinine) were continuous variables. Logistic analyses were adjusted for a reduced number of study variables (maternal age and racial/ethnic group) due to the small number of adverse birth outcomes in the dataset. The association between the season of conception and the odds of LBW or PTB was also examined using logistic regression and comparison of sequential three-month intervals with the remainder of the year. The “season of conception” was determined by estimating the date that pregnancy began, calculated as the number of weeks of gestation multiplied by 7 days per week, and counted back from the infant’s day of birth. Mann-Whitney and chi-square tests were used to evaluate whether the characteristics of the study subjects included in the analyses were similar to the characteristics of the subjects excluded due to missing data for model covariates.

## 3. Results

Two racial/ethnic groups (African-American: 46% and Caribbean/West Indian: 39%) accounted for the majority of the study population (Table 1).

The frequency of fish consumption during pregnancy was high, with 15% of the population reporting consumption several times per week, while the prevalence of alcohol and tobacco use was low (4% and 3%, respectively). Even after coding species consumed into “low”, “high” and “extremely high” mercury exposure levels based on species ranking by the NYC Department of Health and Mental Hygiene [47], we did not have sufficient sample size to include type of species consumed into our models. We did, however, find that some participants were consuming fish high in mercury such as tuna and shark. The prevalence of alcohol and tobacco use was low (4% and 3%, respectively). Nineteen percent of neonates were born preterm (<37 weeks) and 14% were LBW (<2500 g). Median, 25th and 75th percentiles for cord blood and creatinine-corrected urinary Hg are reported in Table 2.

**Table 1:** Study population characteristics.

Participant Characteristics	*N* (Percent)	Mean Infant Birthweight (Grams) (SD)	Mean Number of Weeks Gestation (SD)
*Race/Ethnicity*			
African-American	73 (46)	3006 (546)	37.6 (2.2)
Caribbean/West Indian	62 (39)	3104 (602)	37.9 (2.2)
From African Continent (4), Latino/Hispanic (13) & Other (5)	22 (14)	3120 (476)	38.0 (1.6)
Did not answer	2 (1)	3673 (237)	39.5 (0.7)
*Age group*			
Less than 25 year	61 (38)	3133 (469)	38.2 (2.0)
25 to 29 year	37 (23)	3001 (579)	37.9 (2.1)
30 to 34 year	39 (25)	3037 (620)	37.5 (2.4)
35 and over	22 (14)	3059 (621)	37.1 (2.0)
*Educational attainment*			
Some high school or less	36 (23)	3052 (541)	37.7 (2.2)
High school certificate	50 (31)	3028 (602)	37.8 (2.2)
Technical school, some college or more	73 (46)	3104 (545)	37.8 (2.1)
*Live with spouse/Partner*			
No	81 (51)	3057 (596)	37.7 (2.3)
Yes	77 (48)	3080 (527)	37.9 (2.0)
Did not answer	1 (1)	3110	38
*Frequency of fish intake during this pregnancy*			
Almost never or never	54 (34)	3019 (451)	37.7 (1.9)
1–3 times per month	58 (36.5)	3117 (555)	37.9 (2.2)
4–7 times per month	23 (14.5)	3122 (404)	38.2 (1.6)
Several times per week	24 (15)	3013 (865)	37.3 (2.9)
*Number of dental amalgams*			
None	85 (53)	3100 (523)	37.9 (1.9)
1 to 3	40 (25)	2998 (692)	37.3 (2.7)
4 to 6	25 (16)	3097 (499)	38.1 (2.0)
7 or more	8 (5)	3117 (314)	38.5 (1.7)
Did not answer	1 (1)	2120	36
*Born outside the United States*			
No	84 (53)	3025 (524)	37.7 (2.1)
Yes	75 (47)	3117 (598)	37.9 (2.2)
*Special product use*			
No	147 (92)	3067 (568)	37.8 (2.2)
Yes	9 (6)	3198 (422)	38.1 (1.8)
Did not answer	3 (2)	2753 (558)	38.0 (2.0)
Visited botanica * during pregnancy			
No	150 (94)	3065 (555)	37.8 (2.2)
Yes	8 (5)	3248 (591)	38.6 (0.7)
Did not answer	1 (1)	2120	36
*Alcohol use*			
No	151 (95)	3084 (556)	37.8 (2.2)
Yes	6 (4)	2656 (624)	37.2 (1.9)
Did not answer	2 (1)	3170 (431)	38 (0)
*Tobacco use*			
No	152 (96)	3074 (565)	37.8 (2.2)
Yes	5 (3)	2872 (476)	37.6 (1.7)
Did not answer	2 (1)	3170 (431)	38 (0)
*Season of conception*			
Spring	43 (27)	3143.3(503.7)	38.3 (1.8)
Summer	56 (35)	3109.5 (573.1)	37.7 (2.1)
Fall	33 (21)	3066.9 (527.4)	37.6 (1.9)
Winter	27 (17)	2867.3 (636.4)	37.3 (2.7)
*Birth weight*			
Less than 2500 g	23 (14)	2132 (360)	34.8 (2.9)
2500 g and over	136 (86)	3227 (414)	38.3 (1.5)
*Term of birth*			
Preterm (less than 37 weeks)	30 (19)	2436 (616)	34.5 (2.2)
Term (37 to 42weeks)	129 (81)	3216 (431)	38.6 (1.2)

* A *botanica* is defined as a retail store that sells folk medicine, religious candles, and other products regarded as magical or alternative medicine.

A significant number of respondents were missing data for cord blood Hg (92 observations) or urinary Hg (11 observations). Almost all (98.5%) of cord blood Hg levels and 82.7% of urinary Hg levels were above the method LOD. There was a significant positive correlation between maternal urinary Hg and cord blood Hg (*r =* 0.47, 95% CI 0.34–0.60, *n =* 75) [45]. Caribbean/West Indian women and neonates had the highest cord blood and maternal urinary Hg levels (2.23 µg/L and 0.48 µg/g, respectively), but they were not significantly different from African-American, African-continent or Latina women. LBW neonates did not significantly differ in cord blood or maternal urinary Hg levels compared to neonates weighing over 2500 g (1.70 µg/L and 0.39 µg/g compared to 1.96 µg/L and 0.38 µg/g, *p >* 0.05). Similarly, cord blood or maternal urinary Hg levels did not differ by timing of birth (1.50 µg/L and 0.45 µg/g (PTB) compared to 1.98 µg/L and 0.35 µg/g (term birth group), *p >* 0.05). Maternal urinary Hg levels were lowest in the summer and these findings were statistically significantly different from the fall (*p =* 0.01). When the observations were restricted to only those observations included in the LBW and PTB seasonal models, no significant seasonal difference in maternal urinary Hg occurred (*p =* 0.06, data not shown). We observed no increase in the odds of LBW or PTB associated with either neonate cord blood Hg or maternal urinary Hg (Refer to Table 3). There was no association of LBW or PTB associated with neonate cord blood Hg or maternal urinary Hg when stratified by season (data not shown).

**Table 2:** Cord Blood Hg and Creatinine Corrected Maternal Urinary Hg.

Participant Characterstics	Cord Blood Hg (µg/L)					Urinary Hg (µg/g Creatinine)			
	*N*		Median	[Q1, Q3] ^a^	*p*-Value ^b^	*N*	Median	[Q1, Q3]	*p*-Value ^b^
*Race/Ethnicity*									
African-American		29	1.49	[0.9, 2.64]	0.10	63	0.35	[0.11, 0.78]	0.22
Caribbean/West Indian		26	2.23	[1.78, 4.20]		59	0.48	[0.16, 0.83]	
From African continent, Latino/Hispanic & Other		11	1.44	[0.8, 5.02]		22	0.28	[0.07, 0.63]	
*Neonate birth weight*									
Less than 2500 g		10	1.70	[1.30, 2.04]	0.63	21	0.39	[0.08, 0.67]	0.60
2500g and over		57	1.96	[1.15, 3.65]		125	0.38	[0.14, 0.80]	
*Week of gestation at birth*									
Less than 37		11	1.50	[1.30, 2.04]	0.19	28	0.45	[0.19, 0.74]	0.69
37 to 42		56	1.98	[1.20, 4.70]		118	0.35	[0.12, 0.79]	
Season of conception									
Spring		21	2.27	[1.11, 4.90]	0.20	42	0.33	[0.14, 0.74]	**0.04**
Summer		14	1.47	[0.81, 1.80]		53	0.28	[0.07, 0.61]	
Fall		14	2.13	[1.37, 3.65]		28	0.66	[0.25, 0.89]	
Winter		18	1.91	[1.25, 4.95]		23	0.44	[0.26, 0.80]	

^a^ Q1 = 25th percentile, Q3 = 75th percentile; ^b^ Kruskal-Wallis ANOVA *p*-value.

Similarly, there was no significant change in birth weight, body length, or head circumference with changes in neonate cord blood or maternal urinary Hg (Table 4). Adjustment for fish consumption (data not shown) in linear and logistic models did not change results considerably.

The overall findings did not change when the analysis was stratified by presence or absence of dental amalgams (data not shown).

**Table 3:** Association of cord blood Hg and urinary Hg with preterm birth and low birth weight (LBW).

Logistic Regressions ^a^	Odds Ratio	95% Confidence Interval (CI)	*p*-Value ^b^
*LBW*			
Cord blood Hg (*n =* 66)	1.07	[0.72, 1.61]	0.73
Creatinine-corrected urine Hg (*n =* 144)	0.51	[0.14, 1.87]	0.27
*PTB*			
Cord blood Hg (*n =* 66)	0.65	[0.38, 1.12]	0.04
Creatinine-corrected urine Hg (*n =* 144)	0.78	[0.38, 1.59]	0.48

^a^ Logistic regressions were adjusted for maternal age group and racial ethnic group. LBW model included term of birth. ^b^Likelihood ratio test p-values. Models including either cord blood Hg or creatinine-corrected urine Hg did not provide better fit than reduced models not containing either cord blood Hg or creatinine-corrected urine Hg variable (Likelihood ratio test *p >* 0.05) except for the PTB cord blood Hg model (LR *p =* 0.03); however, all women (*n =* 10) who reported consuming fish 4–7 times per month and who had neonate cord blood Hg measurements had term deliveries and were dropped from the logistic regression analysis for PTB.

**Table 4:** Association of cord blood Hg and urinary Hg with neonate birth weight, head circumference and length.

Linear Regressions	β Coefficients ^a^	95% CI	*p*-Value ^b^
*Birth weight* (*in grams*)			
Cord blood Hg (*n =* 64)	4.42	[−7.38, 16.22]	<0.01
Creatinine-corrected urine Hg (*n =* 140)	−1.23	[−7.35, 4.88]	<0.01
*Head Circumference* (*cubed*, *in cm^3^*)			
Cord blood Hg (*n =* 64)	61.16	[−66.25, 188.57]	0.05
Creatinine-corrected urine Hg (*n =* 137)	3.63	[−66.84, 74.10]	<0.01
*Length* (*squared*, *in cm^2^*)			
Cord blood Hg (*n =* 62)	−0.24	[−10.46, 9.98]	0.16
Creatinine-corrected urine Hg (*n =* 133)	−1.74	[−6.20, 2.71]	<0.01

Linear regressions were adjusted for age group, education attainment, racial/ethnic group, and living with partner/ spouse. Birth weight models also included term of birth. ^a^ β–coefficients represent the change in outcome variable (birth weight (g), head circumference (cm^3^), and length (cm^2^)) with each 10% increase in cord blood or maternal urine Hg; ^b^ Likelihood ratio test *p*-values.

Mann-Whitney and chi-square tests were used to evaluate whether the characteristics of the study subjects included in the multivariate analysis were similar to the characteristics of the subjects excluded due to missing data for model covariates. Refer to Appendix Tables A1–A8. For the LBW and PTB logistic regressions which included the cord blood mercury variable, excluded subjects were more likely than included subjects to live with a spouse or partner (55% and 39%, respectively, *p =* 0.05) and report alcohol use (6.5% and 0%, respectively, *p =* 0.03). For the linear regressions that included the cord blood mercury variable, none of the study subjects included in the analysis reported alcohol use, which was statistically significantly different than the excluded population (0% and 6.5%, respectively, *p =* 0.04). For the logistic and linear regressions that included the maternal urinary mercury variable, the excluded subject group included a higher percentage of African-American women in comparison to Caribbean/West Indian women (for example, 77% African-American and 23% Caribbean/West Indian in the excluded group compared to 44% and 41%, respectively for the subjects included in the PTB and LBW analysis, *p =* 0.02). There were no other statistical differences between the groups in regards to participant characteristics.

**Figure 1:**
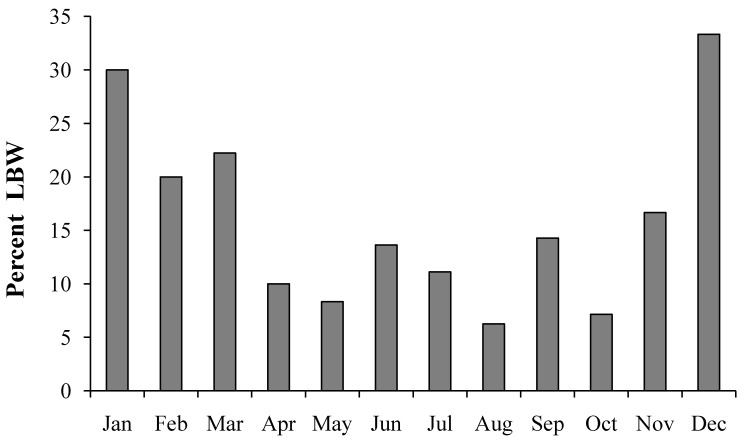
Percent of neonates with low birthweight by month of conception.

Figure 1 shows higher percentages of LBW births during the months of December through March. Odds ratios for season of conception derived based on 3 month groupings is provided in Table 5. The largest OR is found in the three-month aggregate of December, January, and February adjusted for maternal age and racial/ethnic group were OR: 7.52 [95% CI 1.65, 34.29] (Table 5).

The association of season of conception and PTB was similar, but not significant (winter versus all other seasons OR: 1.33 [95% CI 0.46, 3.80]).

**Table 5:** Association of season of conception with adverse birth outcomes.

Season of Conception	OR	95% CI	*p*-Value ^a^
*LBW*			
Winter (December, January, February) *vs*. all other months	**7.52**	[1.65, 34.29]	*p =* 0.01
Spring (March, April, May) *vs*. all other months	0.59	[0.15, 2.29]	*p =* 0.44
Summer (June, July, August) *vs*. all other months	0.75	[0.21, 2.61]	*p =* 0.65
Fall (September, October, November) *vs*. all other months	0.42	[0.09, 1.89]	*p =* 0.24
*Preterm Birth*			
Winter (December, January, February) *vs*. all other months	1.33	[0.46, 3.80]	*p =* 0.60
Spring (March, April, May) *vs*. all other months	1.01	[0.39, 2.62]	*p =* 0.98
Summer (June, July, August) *vs*. all other months	0.62	[0.25, 1.56]	*p =* 0.30
Fall (September, October, November) *vs*. all other months	1.39	[0.53, 3.66]	*p =* 0.51

LBW models were adjusted for term of birth, maternal age group and race/ethnicity. PTB models were adjusted for maternal age group and race/ethnicity. Dates are coded as Spring (1 March–31 May), Summer (1 June–31 August), Fall (1 September–31 November ) and Winter (1 December–28/9 February). *N =* 157 for all models, there were 23 LBW neonates and 30 PTB neonates in total. ^a^ Likelihood ratio test *p*-values. Models containing the seasonal variable did not provide a significantly better fit than the reduced models (Likelihood ratio test *p >* 0.05) except for the LBW winter model (LR *p =* 0.01).

## 4. Discussion

PTB and LBW disproportionately affect minority populations and result in acute and chronic health impacts. Previous studies report that cultural practices may increase exposure to Hg through dietary consumption [48]. Fish consumption habits reported in this study, such as higher reported consumption in certain racial/ethnic groups, were in line with those reported in McKelvey *et al*. (2011) [49] in the NYC population. This study found no association between neonate cord blood Hg or maternal urinary Hg levels and LBW or continuous anthropometric outcomes, and no association of maternal urinary Hg with PTB. This could suggest that though these women were exposed to Hg, fish consumption had a beneficial effect on gestation length as seen in prior studies [50,51], or could indicate sampling error due to the small sample size. The cord blood Hg levels found in this study were lower than in other studies reporting an association between decreased birth weight with increased Hg exposure. In a study of women exposed to Hg through consumption of traditional diets in Greenland, Foldspang and Hanson (1990) [52] reported decreased birth weight with increasing maternal and neonate cord blood levels, but neonate cord blood Hg levels ranged from 2 to 136 µg/L, with a mean of 21.0 µg/L [52]. Consumption habits and consequent MeHg levels from this population certainly cannot be considered within the “normal” range of most fish-consuming populations, such as in most areas of the USA [48]. Ramon *et al* (2009) [33] also reported a negative association between cord blood Hg and mean birth weight, but maternal fish consumption was also much higher (only 1.6% of women reported rarely or never eating fish compared to 34% in this cohort) and 72% of the neonates had cord blood Hg levels >5.8 µg/L [33]. Maternal urinary Hg in this study ranged from 0.24 to 3.50 µg/g with a geometric mean of 0.32 µg/g and 95th percentile of 1.9 µg/g. In a comparison study, the population-weighted geometric mean and 95th percentile of 0.63 and 0.83 µg/g, and 1.13 and 1.45 µg/g, respectively, was reported in Non-Hispanic Blacks and Caribbean-born Non-Hispanic Blacks in New York City [49]. Thus findings from our study are in line with levels found in large population-based studies in the USA.

Differences in Hg levels may be attributed to cultural differences in quantity of meal or type of fish consumed as well as local availability of various types of fish. The lack of association between total blood Hg exposure, mainly MeHg, and birth outcomes in this study is consistent with other studies of low-level Hg exposure that have also have found no association [18]. Sample size limitations could contribute to lack of association found, as well as use of maternal urinary Hg in our birth outcomes models, a less accurate measure of MeHg exposure than total Hg in blood [53] which was the main measure of exposure used in comparable studies examining birthweight. Accounting for varying levels of fish consumption, which has been done in prior studies, had no measureable effect on model results.

Our study revealed increased odds of LBW neonates for pregnancies that began in December, January and February. In an Australian study, Ford (2011) [54] found a similar association of small for gestational age neonates and season of conception (2 × 2 contingency test, *p =* 0.01). Of 401 live births born to 585 couples enrolled in a prospective study, 11 of the neonates had birth weights lower than the 3rd percentile of national weights. Six of these neonates were conceived in winter, while 5 were conceived in spring [54]. Other studies that examine the association of birth weight with season of birth have found elevated rates of LBW in summer and autumn compared to winter and spring [19], which would be consistent with a season of conception in fall and winter.

This study did not find an association between the season of conception and PTB. In contrast, Bodnar and Simhan (2008) [55] found that the peak prevalence of PTB in a retrospective cohort study of 82,213 singleton livebirths. The present study is not sufficiently powered to identify small differences in prevalence of PTB as identified in the Bodnar and Simhan study [55]. Season serves as a proxy for geophysical conditions, environmental exposures, and psychosocial events such as annual religious holidays [56,57]. The observed association may also be due to lower Vitamin D uptake during winter months. Season can also serve as a proxy for exposure to air pollutants that vary, particularly those related to petroleum products and vehicle exhaust such as 1,3 butadiene, benzene, xylene and cadmium, increase in the winter [58]. Additional combustion byproducts of fuel consumption released during winter could include PM and other possible co-varying pollutants such as SO_2_, both of which have been associated with effects on birthweight [59,60]. Levels of indoor and outdoor non-volatile polycyclic aromatic hydrocarbons (PAHs) have been shown to increase during the heating season in New York City [60] and levels of ambient volatile organic compounds benzo[a]pyrene, toluene, ethylbenzene, and xylene were higher in winter in a Camden, New Jersey study [61]. The increased incidence of infectious diseases in the winter also cannot be ruled out. In contrast, several studies show a seasonal effect of elevated blood lead levels in summer months due to increased play in outdoor contaminated areas, increased hand to mouth activity, and possibly even physiologic factors [62].

This study is the first to examine exposure to Hg and season of conception with risk of LBW and PTB birth in this New York City community. In a prior study in this same population, Lijinian *et al* (1997) [63] found an association between preterm labor and high heat-humidity index, stressing the need for further study of seasonality effects on timing of birth. Seasonal variability in birth weight has been associated with temperature in previous studies [64,65]. Strengths of this study include the prospective study design and the inclusion of a population at high risk for adverse birth outcomes and increased fish consumption. The use of individual-level measures of maternal and neonate Hg exposures removes bias by providing an independent level of measurement that is not subject to recall bias or misclassification error that can occur if exposure is solely determined by a diet history. Medical records provided neonate anthropometric data as well as immigration history for the non-US born women. One of the limitations of the study is convenience sampling, which may have resulted in selection bias. It is possible that that the lack of an association between Hg exposure and adverse birth outcomes is due to beneficial actions of ω-3 fatty acids available through fish consumption. Though levels of ω-3 fatty acids were not measured directly in these women, we adjusted models for fish consumption (data not shown) to account for possible nutritional benefits and as a proxy for healthy lifestyle effects on birthweight. Adjustment for fish consumption did not measurably change model results. The measures of prenatal exposure were limited to two different time points, and thus could have led to inaccurate characterization of exposure. Additionally, the small sample size may have limited our ability to detect an association. The season of conception may have been misclassified during calculation of the date of conception, as estimation of gestational age using either a woman’s recall of the first day of her last menstrual period, or ultra-sound dating that may be inaccurate [66,67]. Classifying pregnancies that began within a few days of the end of the season may have biased the association, since the majority of the beginning of the first trimester would have occurred during the adjacent season. Other parameters that may have influenced birth weight such as parity, maternal height, weight and body mass index [68,69] were unavailable and were not included in regression models. Neighborhood-level effects such as the level of neighborhood organization, ethnic density and other psychosocial factors have been associated with PTB and/or LBW but were not examined in this study [70,71,72,73]. Since season of conception and season of birth are not independent, seasonal exposures during other seasons may be driving the association seen in this study.

In conclusion, this study is consistent with others that do not show an association between prenatal Hg exposure and adverse birth outcomes. Further examination of the factors that may influence the seasonal association with LBW is needed.

## Appendix

**Table A1:** Comparison of included versus excluded cases for cord blood Hg and LBW and PTB models. CI= included in model, CE = excluded.

Participant Characteristics	CI (66)	CE (93)	*p*-Value
*Race/Ethnicity*			*0.47*
African-American	29	44	
Caribbean/West Indian	26	36	
From African Continent, Latino/Hispanic & Other	11	11	
Did not answer		2	
*Age group*			*0.72*
Less than 25 year	27	34	
25 to 29 year	14	23	
30 to 34 year	16	23	
35 and over	9	13	
*Educational attainment*			*0.27*
Some high school or less	17	19	
High school certificate	22	28	
Technical school, some college or more	27	46	
*Live with spouse/partner*			***0.05***
No	40 (61%)	41 (44%)	
Yes	26 (39%)	51 (55%)	
Did not answer	0	1	
*Frequency of fish intake during this pregnancy*			*0.55*
Almost never or never	21	33	
1–3 times per month	24	34	
4–7 times per month	10	13	
Several times per week	11	13	
*Number of dental amalgams*			*0.92*
None	35	50	
1 to 3	18	22	
4 to 6	7	18	
7 or more	5	3	
Did not answer	1		
*Born outside the United States*			*0.21*
No	31	53	
Yes	35	40	
*Special product use*			*0.18*
No	59	88	
Yes	6	3	
Did not answer	1	2	
*Visited botanica during pregnancy*			*0.21*
No	62	88	
Yes	3	5	
Did not answer	1		
No	66	85	
Yes	0 (0%)	6 (6.5%)	
Did not answer		2	
*Tobacco use*			
No	66	86	*0.053*
Yes	0 (0%)	5 (5.4%)	
Did not answer		2	
*Birth weight*			*0.84*
Less than 2500 g	10	13	
2500 g and over	56	80	
*Term of birth*			*0.55*
Preterm (less than 37 weeks)	11	19	
Term (37 to 42weeks)	55	74	

**Table A2:** Comparison of included versus excluded cases for urine Hg and LBW and PTB models. UI = included in model, UE = excluded.

Participant Characteristics	UI (144)	UE (15)	*p*-Value
*Race/Ethnicity*			***0.02***
African-American	63 (44%)	10 (77%)	14%exc
Caribbean/West Indian	59 (41%)	3 (23%)	5%exc
From African Continent, Latino/ Hispanic & Other	22	0	
Did not answer		2	
*Age group*			*0.60*
Less than 25 year	54	7	
25 to 29 y year	35	2	
30 to 34 year	34	5	
35 and over	21	1	
*Educational attainment*			*0.11*
Some high school or less	34	2	
High school certificate	47	3	
Technical school, some college or more	63	10	
*Live with spouse/partner*			*0.36*
No	75	6	
Yes	68	9	
Did not answer	1	0	
*Frequency of fish intake during this pregnancy*			*0.56*
Almost never or never	51	3	
1–3 times per month	50	8	
4–7 times per month	21	2	
Several times per week	22	2	
*Number of dental amalgams*			*0.97*
None	77	8	
1 to 3	37	3	
4 to 6	24	1	
7 or more	6	2	
Did not answer		1	
*Born outside the United States*			*0.56*
No	75	9	
Yes	69	6	
*Special product use*			*0.50*
No	135	12	
Yes	9	0	
Did not answer		3	
*Visited botanica during pregnancy*			*0.37*
No	62	88	
Yes	3	5	
Did not answer	1	0	
*Alcohol use*			*0.55*
No	137	14	
Yes	5	1	
Did not answer	2	0	
*Tobacco use*			*0.46*
No	137	15	
Yes	5	0	
Did not answer	2		
*Birth weight*			*0.90*
Less than 2500 g	21	2	
2500 g and over	123	13	
*Term of birth*			*0.57*
Preterm (less than 37 weeks)	28	2	
Term (37 to 42weeks)	116	13	

**Table A3:** Comparison of included *versus* excluded cases for linear regression of birthweight and urinary Hg. BWLRUI = included in model. BWLRUE = excluded.

Participant Characteristics	BWLRUI	BWLRUE	*p*-Value
*Race/Ethnicity*			***0.02***
African-American	61	12	
Caribbean/West Indian	57	5	
From African Continent, Latino/Hispanic & Other	22	0	
Did not answer		2	
*Age group*			*0.74*
Less than 25 year	54	7	
25 to 29 year	34	3	
30 to 34 year	32	7	
35 and over	20	2	
*Educational attainment*			*0.14*
Some high school or less	33	3	
High school certificate	46	4	
Technical school, some college or more	64	12	
*Live with spouse/partner*			*0.91*
No	72	9	
Yes	68	9	
Did not answer		1	
*Frequency of fish intake during this pregnancy*			*0.32*
Almost never or never	50	4	
1–3 times per month	49	9	
4–7 times per month	21	2	
Several times per week	20	4	
*Number of dental amalgams*			*0.87*
None	75	10	
1 to 3	35	5	
4 to 6	24	1	
7 or more	6	2	
Did not answer		1	
*Born outside the United States*			*0.64*
No	73	11	
Yes	67	8	
*Special product use*			*0.43*
No	129	18	
Yes	9	0	
Did not answer	2	1	
*Visited botanica during pregnancy*			*0.30*
No	132	18	
Yes	8	0	
Did not answer		1	
*Alcohol use*			*0.73*
No	133	18	
Yes	5	1	
Did not answer	2		
*Tobacco use*			*0.40*
No	133	19	
Yes	5	0	
Did not answer	2		
*Birth weight*			*0.86*
Less than 2500 g	20	3	
2500 g and over	120	16	
*Term of birth*			*0.80*
Preterm (less than 37 weeks)	26	4	
Term (37 to 42weeks)	114	15	

**Table A4:** Linear regression model of birthweight and cord blood Hg. BWLRCI = included in model, BWLRCE = excluded.

Participant Characteristics	BWLRCI	BWLRCE	*p*-Value
*Race/Ethnicity*			*0.43*
African- American	28	45	
Caribbean/West Indian	25	37	
From African Continent, Latino/Hispanic & Other	11	11	
Did not answer		2	
*Age group*			*0.45*
Less than 25 year	27	34	
25 to 29 year	14	23	
30 to 34 year	15	24	
35 and over	8	14	
*Educational attainment*			*0.23*
Some high school or less	17	19	
High school certificate	21	29	
Technical school, some college or more	26	47	
*Live with spouse/partner*			*0.09*
No	38	43	
Yes	26	51	
Did not answer		1	
*Frequency of fish intake during this pregnancy*			*0.92*
Almost never or never	21	33	
1–3 times per month	24	34	
4–7 times per month	10	13	
Several times per week	9	15	
*Number of dental amalgams*			*0.95*
None	34	51	
1 to 3	17	23	
4 to 6	7	18	
7 or more	5	3	
Did not answer	1		
*Born outside the United States*			*0.22*
No	30	54	
Yes	34	41	
*Special product use*			*0.16*
No	56	91	
Yes	6	3	
Did not answer	2	1	
*Visited botanica during pregnancy*			*0.89*
No	60	90	
Yes	3	5	
Did not answer	1	0	
*Alcohol use*			
No	64	87	***0.04***
Yes	0	6	
Did not answer		2	
*Tobacco use*			
No	64	88	*0.06*
Yes	0	5	
Did not answer		2	
*Birth weight*			*0.73*
Less than 2500 g	10	13	
2500 g and over	54	82	
*Term of birth*			*0.40*
Preterm (less than 37 weeks)	10	20	
Term (37 to 42weeks)	54	75	

**Table A5:** Linear regression model of head circumference and cord blood Hg. HCLRCI = included in model, HCLRCE = excluded.

Participant Characteristics	HCLRCI	HCLRCE	*p*-Value
*Race/Ethnicity*			*0.42*
African-American	28	45	
Caribbean/West Indian	25	37	
From African Continent, Latino/Hispanic & Other	11	11	
Did not answer			
*Age group*			*0.45*
Less than 25 y	27	34	
25 to 29 y	14	23	
30 to 34 y	15	24	
35 and over	8	14	
*Educational attainment*			*0.23*
Some high school or less	17	19	
High school certificate	21	29	
Technical school, some college or more	26	47	
*Live with spouse/partner*			*0.09*
No	38	43	
Yes	26	51	
Did not answer		1	
*Frequency of fish intake during this pregnancy*			*0.91*
Almost never or never	21	33	
1–3 times per month	34	34	
4–7 times per month	10	13	
Several times per week	9	15	
*Number of dental amalgams*			*0.95*
None	34	51	
1 to 3	17	23	
4 to 6	7	18	
7 or more	5	3	
Did not answer	1		
*Born outside the United States*			*0.22*
No	30	54	
Yes	34	41	
*Special product use*			*0.16*
No	56	91	
Yes	6	3	
Did not answer	2	1	
*Visited botanica during pregnancy*			*0.89*
No	60	90	
Yes	3	5	
Did not answer	1		
*Alcohol use*			***0.04***
No	64	87	
Yes	0	6	
Did not answer		2	
*Tobacco use*			
No	64	88	*0.06*
Yes	0	5	
Did not answer		2	
*Birth weight*			*0.73*
Less than 2500 g	10	13	
2500 g and over	54	82	
*Term of birth*			*0.39*
Preterm (less than 37 weeks)	10	20	
Term (37 to 42weeks)	54	75	

**Table A6:** Linear regression model of head circumference and urinary Hg. HCLRUI = included in model, HCLRUE = excluded.

Participant Characteristics	HCLRUI	HCLRUE	*p*-Value
*Race/Ethnicity*			
African-American	59	14	**0.02**
Caribbean/West Indian	57	5	
From African Continent, Latino/Hispanic & Other	21	1	
Did not answer		2	
*Age group*			*0.54*
Less than 25 year	53	8	
25 to 29 year	34	3	
30 to 34 year	31	8	
35 and over	19	3	
*Educational attainment*			*0.53*
Some high school or less	31	5	
High school certificate	45	5	
Technical school, some college or more	61	12	
*Live with spouse/partner*			*0.72*
No	71	10	
Yes	66	11	
Did not answer		1	
*Frequency of fish intake during this pregnancy*			*0.36*
Almost never or never	49	5	
1–3 times per month	48	10	
4–7 times per month	20	3	
Several times per week	20	4	
*Number of dental amalgams*			*0.44*
None	72	13	
1 to 3	35	5	
4 to 6	24	1	
7 or more	6	2	
Did not answer		1	
*Born outside the United States*			*0.53*
No	71	13	
Yes	66	9	
*Special product use*			*0.96*
No	127	20	
Yes	8	1	
Did not answer	2	1	
*Visited botanica during pregnancy*			*0.26*
No	129	21	
Yes	8	0	
Did not answer		1	
*Alcohol use*			*0.85*
No	130	21	
Yes	5	1	
Did not answer			
*Tobacco use*			*0.36*
No	130	22	
Yes	5	0	
Did not answer			
*Birth weight*			*0.91*
Less than 2500 g	20	3	
2500 g and over	117	19	
*Term of birth*			*0.62*
Preterm (less than 37 weeks)	25	5	
Term (37 to 42weeks)	112	17	

**Table A7:** Linear regression model of neonate length and urinary Hg. LLRUI = included in model, LLRUE = excluded.

Participant Characteristics	LLRUI	LLRUE	*p*-Value
*Race/Ethnicity*			***0.04***
African- American	57	16	
Caribbean/West Indian	56	3	
From African Continent, Latino/Hispanic & Other	20	2	
Did not answer		2	
*Age group*			*0.86*
Less than 25 year	50	11	
25 to 29 year	32	5	
30 to 34 year	33	6	
35 and over	18	4	
*Educational attainment*			*0.26*
Some high school or less	31	5	
High school certificate	44	6	
Technical school, some college or more	58	15	
*Live with spouse/partner*			*0.72*
No	69	12	
Yes	64	13	
Did not answer		1	
*Frequency of fish intake during this pregnancy*			*0.52*
Almost never or never	48	6	
1–3 times per month	45	13	
4–7 times per month	20	3	
Several times per week	20	4	
*Number of dental amalgams*			*0.47*
None	69	16	
1 to 3	36	4	
4 to 6	23	2	
7 or more	5	3	
Did not answer		1	
*Born outside the United States*			*0.59*
No	69	15	
Yes	64	11	
*Special product use*			*0.46*
No	124	23	
Yes	7	2	
Did not answer	2	1	
*Visited botanica during pregnancy*			*0.79*
No	126	24	
Yes	7	1	
Did not answer		1	
*lcohol use*			*0.26*
No	127	24	
Yes	4	2	
Did not answer	2		
*Tobacco use*			*0.83*
No	127	25	
Yes	4	1	
Did not answer	2		
*Birth weight*			*0.28*
Less than 2500 g	21	2	
2500 g and over	112	24	
*Term of birth*			*0.30*
Preterm (less than 37 weeks)	27	3	
Term (37 to 42weeks)	106	23	

**Table A8:** Linear regression model of neonate length and cord blood Hg. LLRCI = included in model, LLRCE = excluded.

Participant Characteristics	LLRCI	LLRCE	*p*-Value
*Race/Ethnicity*			*0.86*
African-American	29	44	
Caribbean/West Indian	23	39	
From African Continent, Latino/Hispanic & Other	10	12	
Did not answer		2	
*Age group*			*0.49*
Less than 25 year	26	35	
25 to 29 year	13	24	
30 to 34 year	16	23	
35 and over	7	15	
*Educational attainment*			*0.13*
Some high school or less	17	19	
High school certificate	21	29	
Technical school, some college or more	24	49	
*Live with spouse/partner*			*0.09*
No	37	44	
Yes	25	52	
Did not answer		1	
*Frequency of fish intake during this pregnancy*			*0.93*
Almost never or never	21	33	
1–3 times per month	22	36	
4–7 times per month	10	13	
Several times per week	9	15	
*Number of dental amalgams*			*0.83*
None	33	52	
1 to 3	17	23	
4 to 6	7	18	
7 or more	4	4	
Did not answer			
*Born outside the United States*			*0.57*
No	31	53	
Yes	31	44	
*Special product use*			*0.41*
No	55	92	
Yes	5	4	
Did not answer	2	1	
*Visited botanica during pregnancy*			*0.95*
No	58	92	
Yes	3	5	
Did not answer	1		
*Alcohol use*			
No	62	89	***0.04***
Yes	0	6	
Did not answer		2	
*Tobacco use*			*0.07*
No	62	90	
Yes	0	5	
Did not answer		2	
*Birth weight*			*0.63*
Less than 2500 g	10	13	
2500 g and over	52	84	
*Term of birth*			*0.77*
Preterm (less than 37 weeks)	11	19	
Term (37 to 42weeks)	51	78	
